# A case of glenohumeral joint impingement caused by a giant secondary synovial osteochondromatosis^☆^

**DOI:** 10.1016/j.ijscr.2024.110527

**Published:** 2024-10-26

**Authors:** Kazuki Hayakawa, Yusuke Kawano, Takashi Kuroiwa, Yukio Nakajima, Katsuji Suzuki, Nobuyuki Fujita

**Affiliations:** a Department of Orthopaedic Surgery, Fujita Health University School of Medicene, Aichi, Japan; b Department of Orthopaedic Surgery, Fujita Health University Okazaki Medical Center, Aichi, Japan

**Keywords:** Synovial osteochondromatosis, Shoulder, Secondary, Glenohumeral joint

## Abstract

**Introduction and importance:**

Synovial osteochondromatosis of the shoulder joint is predominantly primary, characterized by multiple osteochondral fragments, with reports of secondary synovial osteochondromatosis being rare.

**Case presentation:**

The patient, a 48-year-old male, presented to our hospital with right shoulder pain persisting for several months. While there was no significant restriction in the range of motion, pain was noted during horizontal adduction and external rotation in the dependent position. Radiographs and CT scans revealed an osteochondral loose body in the glenohumeral joint and an osteophyte on the anterior margin of the glenoid cavity. A lidocaine test in the glenohumeral joint was positive, suggesting impingement by the loose body, leading to its surgical removal. Arthroscopically, the loose body was grasped and removed from the anterior aspect of the glenohumeral joint. The osteochondral fragment measured approximately 15 mm, with the total length including soft tissue being about 40 mm. Pathological findings indicated a layered arrangement of synovial cells, consistent with secondary synovial osteochondromatosis. Postoperatively, the shoulder pain improved rapidly, and follow-up was concluded six months after surgery.

**Clinical discussion:**

In this case, arthroscopy revealed a Hill-Sachs-like lesion and labral deficiency on the glenoid, suggesting past trauma. However, no bone defect matching the size of the loose body was observed. In secondary synovial osteochondromatosis, small osteochondral fragments can grow with nourishment from the synovium, suggesting the loose body in this case might have similarly enlarged post-trauma.

**Conclusion:**

The shoulder pain caused by a giant secondary synovial osteochondromatosis improved by removing the loose body arthroscopically.

## Highlights

• Shoulder pain caused by a large loose body exceeding 40 mm due to secondary osteochondromatosis.
• Upon performing the surgery using arthroscopy, findings were observed that were thought to be related to past trauma.
• The pathological examination also revealed findings consistent with secondary osteochondromatosis.
• The removal of the loose body under arthroscopy led to a prompt improvement in pain.

## 1 Case report

A 48-year-old male presented with right shoulder joint pain. He had a medical history of diabetes and hypertension and had experienced right shoulder pain since practicing judo in middle school. He noted a clicking sound and severe pain in his right shoulder several months prior while retrieving a cell phone from his left chest pocket, leading him to visit a nearby clinic. Suspected of having a loose body in the glenohumeral joint based on plain radiographs, he was referred to our hospital.

**Fig. 1:**
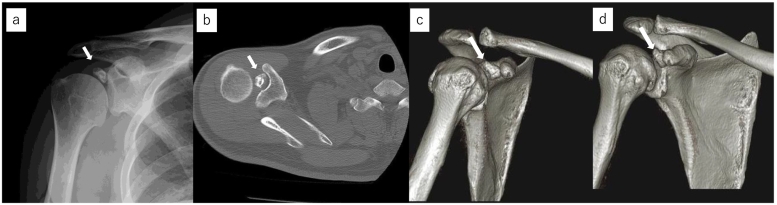
A large loose body was observed within the glenohumeral joint on the plain radiograph and CT images. The loose body is indicated by an arrow. a: The plain radiograph. b: Axial view of plain CT. c: 3D CT imaging revealed the presence of a loose body around the coracoid process. d: Bone spur formation was observed at the anterior edge of the glenoid cavity of the scapula.

**Fig. 2:**
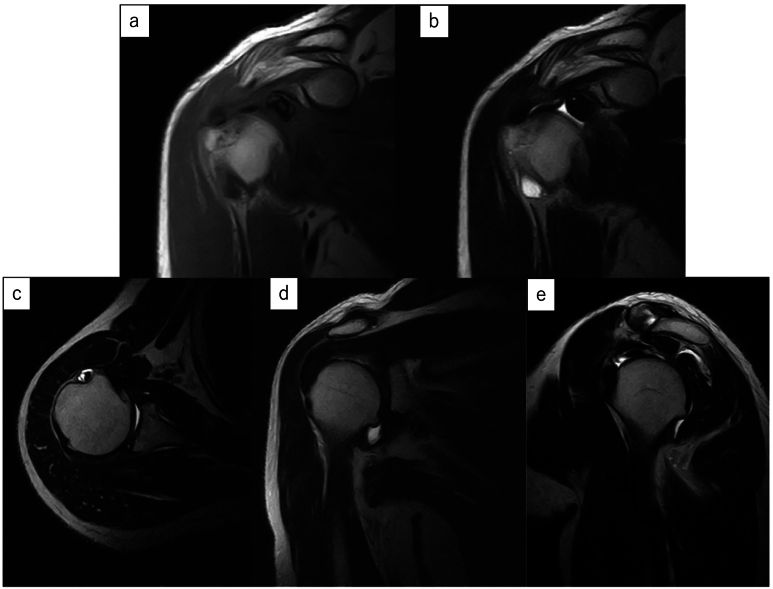
a, b MRI showed a loose body with isointensity in the center and low intensity at the periphery on T1-weighted images, and low intensity on T2-weighted images. a: T1WI: T1-weighted images. b**:** T2WI; T2-weighted images. c, d, e MRI did not reveal any obvious rotator cuff tears. c: Axial view d: Oblique Coronal view e: Oblique Sagittal view.

Upon initial examination, the shoulder joint's active range of motion was 180° in forward elevation, 180° in abduction, 30° in external rotation at the dependent position, and internal rotation reaching the 10th thoracic vertebra. Both the Painful Arc sign and Hawkins sign were positive, and pain was induced during horizontal adduction and external rotation in the dependent position. Plain radiographs and CT scans showed an osteochondral loose body in the suspected glenohumeral joint area, with 3D CT revealing a loose body beneath the coracoid process and osteophyte formation on the anterior margin of the glenoid cavity (Fig. 1). MRI confirmed the presence of a layered bright change foreign body in the glenohumeral joint (Fig. 2a, b), without evidence of a rotator cuff tear (Fig. 2c, d, e). Injection of 1% lidocaine into the glenohumeral joint alleviated pain in provocative positions, suggesting impingement by the intra-articular loose body. Consequently, arthroscopic removal of the loose body was performed.

**Fig. 3:**
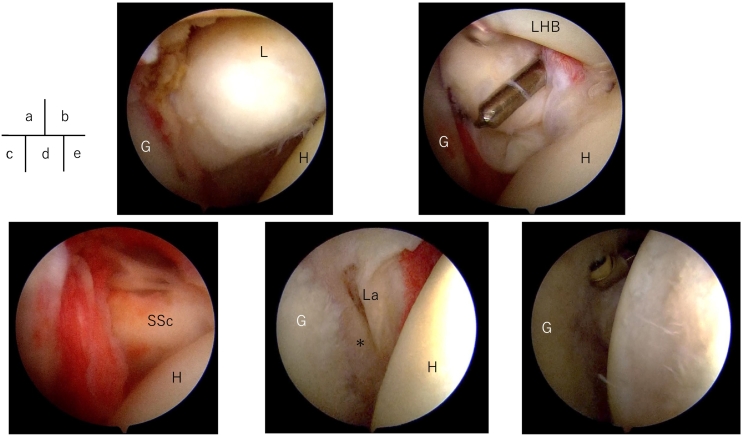
Intraoperative arthroscopic images. a: A large loose body was observed between the glenoid cavity of the scapula and the humeral head. H: Humeral Head, G: Glenoid, L: Loose Body. b: Employ the anterior portal to grasp and extract the loose body using Kocher forceps. LHB: Long head of Biceps. c: Postoperative arthroscopic findings of the shoulder joint immediately after the extraction of the loose body. SSc: subscapuralis muscle. d: Damage to the anterior labrum was observed, along with the presence of an osteophyte formation in the [*] region. e: A bone defect on the posterior-lateral aspect of the humeral head, resembling a Hill-Sachs lesion.

**Fig. 4.**
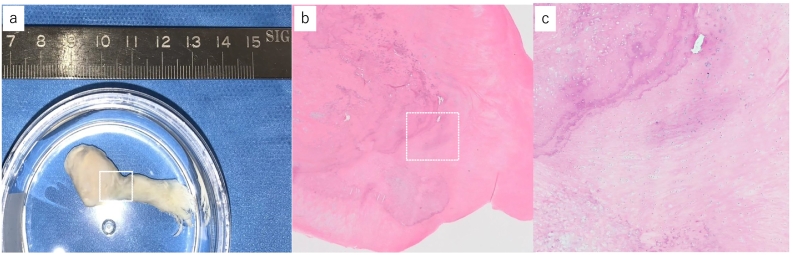
a: The extracted foreign body measured approximately 15 mm in its bony component and about 40 mm when including the suspected ligamentous soft tissue. b: H&E staining findings of the area marked by the rectangle in Fig. 6a. C: Layered arrangement was observed at the indicated location under 40× magnification.

Surgery was conducted under general anesthesia in a beach chair position. Arthroscopy revealed moderate synovitis and a loose body anteriorly within the glenoid cavity, which was pushed towards the joint cavity and removed from the anterior portal (Fig. 3). The osteochondral fragment measured approximately 15 mm in bone and 40 mm including soft tissue resembling the labrum (Fig. 4a). Postoperative radiographs confirmed the removal of the loose body from the glenohumeral joint.

**Fig. 5:**
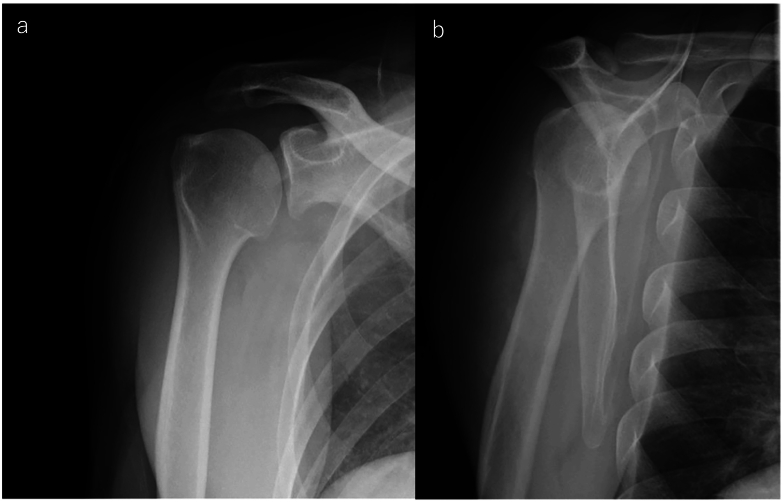
Postoperative shoulder radiographs at six months showed no evidence of a new loose body. a: Anteroposterior view. b: Scapular Y-view.

Pathologically, the osteochondral component showed a layered concentric arrangement, consistent with secondary synovial osteochondromatosis (Fig. 4b, c). The labrum-like soft tissue exhibited degenerative changes and was histologically sparse. Postoperatively, shoulder joint training began after one week of sling immobilization. At two weeks post-surgery, shoulder joint active elevation was 180°, external rotation in the dependent position was 30°, internal rotation reached the 7th thoracic vertebra, and both Painful Arc sign and Hawkins sign were negative. Pain during horizontal adduction and external rotation in the dependent position had disappeared, as had pain induced by internal and external rotation or shoulder abduction movements, and there were no signs of impingement. Six months postoperatively, the patient had no difficulties in daily life, including work, and was discharged from follow-up. Additionally, no loose body was observed on plain radiographs (Fig. 5). The patient provided written consent to submit the manuscript, and the study was approved by our hospital (HM23-357). This report adheres to the SCARE (Surgical CAse REport) criteria for the presentation of surgical case reports [1].

## 2 Discussion

Diseases forming loose bodies in the shoulder joint include synovial osteochondromatosis, osteochondritis dissecans, osteophytes, and joint fractures. In this case, there was no clear traumatic episode, suggesting secondary synovial osteochondromatosis. Synovial osteochondromatosis is classified into primary and secondary forms, with the majority of shoulder cases being primary [2]. Primary synovial osteochondromatosis involves idiopathic synovial chondrometaplasia, whereas secondary synovial osteochondromatosis results from prior conditions like trauma (osteochondral fractures), osteoarthritis, osteochondritis dissecans, or inflammatory diseases, causing small osteochondral fragments to be nourished by synovial fluid and enlarge into osteochondral loose bodies [3]. This case presented labral injury and osteophyte formation on the anterior glenoid margin (Fig. 3d), as well as a Hill-Sachs-like lesion on the posterosuperior humeral head (Fig. 3e), with a history of recurrent shoulder pain since middle school. Given the surgical and pathological findings, secondary synovial osteochondromatosis was diagnosed.

Distinguishing primary from secondary synovial osteochondromatosis involves differences in pathological features [4], proliferating cell nuclear antigen (PCNA) positivity rate in immunological testing [5], and fibroblast growth factor (FGF9) expression [6,7]. Utashima et al. noted that primary cases show nodular synovial cell arrangements, while secondary cases often have layered calcification, suggesting that concentric rings are indicative of secondary synovial osteochondromatosis [4]. In this case, pathological examination showed layered concentric calcification, supporting the diagnosis of secondary synovial osteochondromatosis. Although not used in this case, Saotome et al. reported that in immunological testing of PCNA in surgically removed loose bodies, 100% of primary cases (53/53) were PCNA positive, compared to 45.6% (15/37) of secondary cases, indicating that a negative result suggests secondary synovial osteochondromatosis [8].` Robinson et al. found that cells around primary chondroid nodules expressed PCNA and FGF9, with FGF9 playing a role in cell proliferation, differentiation, and movement necessary for normal cell development and tissue repair [6]. In primary synovial osteochondromatosis, excess FGF9 in the synovial fluid induces chondrogenesis by residual mesenchymal stem cells, suggesting that simple removal of loose bodies might be insufficient. Conversely, secondary synovial osteochondromatosis lacks FGF9 expression, implying a lower recurrence rate post-removal of loose bodies [9]. In this case, since it was considered secondary, follow-up was concluded at six months postoperatively; however, if it had been primary, long-term follow-up over several years would likely have been necessary.

Reports of arthroscopic removal of large osteochondral fragments exceeding 10 mm in the shoulder joint are occasionally seen [10]. Yucel et al. reported a case of synovial chondromatosis following dislocation, with pathological examination but without presentation of histological specimens. In that case, Bankart repair was performed simultaneously due to dislocation. Unlike our case, the osteochondral fragment did not include soft tissues such as ligaments. To our knowledge, reports of large osteochondral loose bodies with soft tissue components in the shoulder are unprecedented.

Several reports exist on secondary synovial osteochondromatosis of the shoulder, [3,8,9,11,12,13], yet these typically involve multiple fragments rather than a single large fragment causing pain. This might be the first English report of shoulder pain due to a single large osteochondral fragment without a clear history of trauma.

In this case, the patient had no memory of significant trauma, yet arthroscopy revealed a Hill-Sachs-like lesion and labral deficiency from 3 to 6 o'clock on the glenoid, suggesting past trauma. However, no bone defect matching the size of the loose body was observed. In secondary synovial osteochondromatosis, small osteochondral fragments can grow with nourishment from the synovium [3,11], suggesting the loose body in this case might have similarly enlarged post-trauma.

## 3 Conclusion

We experienced a case of a giant intra-articular loose body in the shoulder joint, considered secondary synovial osteochondromatosis due to trauma. Arthroscopic removal of the loose body resulted in pain improvement. Sudden shoulder impingement syndrome following past trauma may follow a course similar to this case.

## Consent

Written informed consent was obtained from the patient for publication of this case report and accompanying images. A copy of the written consent is available for review by the Editor-in-Chief of this journal on request.

## Ethical approval

The research related to human use has been complied with all the relevant national regulations, institutional policies and in accordance the tenets of the Helsinki Declaration, and has been approved by the authors' institutional review board or equivalent committee.

## Guarantor

Yusuke Kawano, Co-responding Author.

## Research registration number

1. Name of the registry: Japanese Orthopaedic Association National Registry.
2. Unique identifying number or registration ID: 579628.
3. Hyperlink to your specific registration (must be publicly accessible and will be checked): none.

## Funding

The authors did not receive support from any organization for the submitted work.

## Author contribution

K.Hayakawa and Y.Kawano were mainly involved in writing the paper. Y.Kawano, Y.Nakajima, T.Kuroiwa and K.Suzuki were involved in treating patients, and N.Fujita was in charge of overseeing everything from patient treatment to writing the paper.

## Conflict of interest statement

The authors state no conflict of interest.
